# Domestication Genomics of the Open-Pollinated Scarlet Runner Bean (*Phaseolus coccineus* L.)

**DOI:** 10.3389/fpls.2017.01891

**Published:** 2017-11-15

**Authors:** Azalea Guerra-García, Marco Suárez-Atilano, Alicia Mastretta-Yanes, Alfonso Delgado-Salinas, Daniel Piñero

**Affiliations:** ^1^Posgrado en Ciencias Biológicas, Universidad Nacional Autónoma de México, Ciudad de México, Mexico; ^2^Departamento de Ecología Evolutiva, Instituto de Ecología, Universidad Nacional Autónoma de México, Ciudad de México, Mexico; ^3^Departamento de Ecología de la Biodiversidad, Instituto de Ecología, Universidad Nacional Autónoma de México, Ciudad de México, Mexico; ^4^CONACYT-CONABIO, Comisión Nacional para el Conocimiento y Uso de la Biodiversidad, Ciudad de México, Mexico; ^5^Departamento de Botánica, Instituto de Biología, Universidad Nacional Autónoma de México, Ciudad de México, Mexico

**Keywords:** domestication, genotyping by sequencing, *Phaseolus coccineus*, adaptative variation, population genomics

## Abstract

The runner bean is a legume species from Mesoamerica closely related to common bean (*Phaseolus vulgaris*). It is a perennial species, but it is usually cultivated in small-scale agriculture as an annual crop for its dry seeds and edible immature pods. Unlike the common bean, *P. coccineus* has received little attention from a genetic standpoint. In this work we aim to (1) provide information about the domestication history and domestication events of *P. coccineus*; (2) examine the distribution and level of genetic diversity in wild and cultivated Mexican populations of this species; and, (3) identify candidate loci to natural and artificial selection. For this, we generated genotyping by sequencing data (42,548 SNPs) from 242 individuals of *P. coccineus* and the domesticated forms of the closely related species *P. vulgaris* (20) and *P. dumosus* (35). Eight genetic clusters were detected, of which half corresponds to wild populations and the rest to domesticated plants. The cultivated populations conform a monophyletic clade, suggesting that only one domestication event occurred in Mexico, and that it took place around populations of the Trans-Mexican Volcanic Belt. No difference between wild and domesticated levels of genetic diversity was detected and effective population sizes are relatively high, supporting a weak genetic bottleneck during domestication. Most populations presented an excess of heterozygotes, probably due to inbreeding depression. One population of *P. coccineus* subsp. *striatus* had the greatest excess and seems to be genetically isolated despite being geographically close to other wild populations. Contrasting with previous studies, we did not find evidence of recent gene flow between wild and cultivated populations. Based on outlier detection methods, we identified 24 domestication-related SNPs, 13 related to cultivar diversification and eight under natural selection. Few of these SNPs fell within annotated loci, but the annotated domestication-related SNPs are highly expressed in flowers and pods. Our results contribute to the understanding of the domestication history of *P. coccineus*, and highlight how the genetic signatures of domestication can be substantially different between closely related species.

## Introduction

The scarlet runner bean (*Phaseolus coccineus* L.) is one of the five *Phaseolus* species that were domesticated in Mesoamerica, and it is the third-most economically important, after *P. vulgaris* L. and *P. lunatus* L. The domestication process of this species continues today both in the Americas and Europe, where it was introduced by the Spaniards. One of its main characteristics is its ability to tolerate cooler climates than other *Phaseolus* and up to date it is an important food source for smallholders and indigenous groups in Mexico (Salinas, 1988). Despite the cultural value, economic importance, and agronomic potential of *P. coccineus*, little is known about its domestication history and the genetic variability of its wild and cultivated forms.

Wild *P. coccineus* are perennial climbing plants, occurring mostly at mid-high elevations (1,000–3,000 m.a.s.l.), from northern Mexico (Chihuahua) to Panama (Salinas, 1988). It has 11 pairs of chromosomes and an estimated genome size of 660 Mb (Plant DNA C-values database). Contrasting with the autogamous common bean, the scarlet runner bean is an open-pollinated species. The high morphological diversity of this species has been classified under two subspecies (Freytag and Debouck, 2002): *P. coccineus* subsp. *coccineus* (mostly with red flowers), including 11 wild varieties and the domesticated form, and *P. coccineus* subsp. *striatus* (purple or mauve flowers), conformed by eight wild varieties. No genetic evidence supports these subspecies and varieties, but given the environmental and cultural heterogeneous landscape where *P. coccineus* occurs, it is expected that the species should be genetically structured.

As a cultivated species, *P. coccineus* is currently grown in Mexico, Guatemala, Honduras and Costa Rica, and in lesser degree in South America. In Europe, it is mostly cultivated in the United Kingdom, Netherlands, Italy, and Spain (Rodiño et al., 2006). In Mexico, the scarlet runner bean is cultivated both as a self-sufficiency crop by smallholder farmers (<5 ha) and also commercially for urban areas. Besides its native cultivars, in Mexico there is one breeding line (Blanco Tlaxcala) developed using a multi linear method (Vargas-Vázquez et al., 2012). Feral populations are common, but it is unknown if they originated from hybridization between wild and domesticated populations, or if they escaped from cultivation. Wild, feral and domesticated distributions overlap in Mesoamerica, suggesting that there are plenty of opportunities for gene flow to occur, making the domestication history of *P. coccineus* difficult to disentangle without high resolution genetic markers.

The domestication history of the scarlet runner bean has been explored previously with low resolution molecular markers, and multiple domestication events were suggested. Specifically, chloroplast and nuclear SSRs of *P. coccineus* accessions including European domesticated populations, Mesoamerican landraces and wild samples from Mexico, Guatemala, and Honduras (Angioi et al., 2009; Spataro et al., 2011; Rodriguez et al., 2013) suggest that *P. coccineus* domestication took place in the Guatemala-Honduras area, or that alternatively another domestication event occurred in Mexico followed by extensive hybridization with the cultivated populations from Guatemala and Honduras. However, few Mesoamerican samples were included in these studies, and they focused on European domesticated populations. Phylogenetic analyses including more samples from the wide distribution of *P. coccineus* could bring clues about the number of domestication events that took place in this species. For example, if cultivars are grouped in one monophyletic clade, it would suggest one domestication event.

Another interesting feature of *P. coccineus* domestication history is that similar levels of genetic variation have been reported in wild and cultivated populations (Escalante et al., 1994; Spataro et al., 2011; Rodriguez et al., 2013). This contradicts the population genetics models that predicts a genetic diversity reduction and increased divergence between wild and domesticated forms due to demographic factors and selection at target loci (Meyer and Purugganan, 2013). This pattern has been described in crops like sunflower (∼30%; Renaut and Rieseberg, 2015); soybean (∼30%; Li et al., 2013); maize (∼17%; Hufford et al., 2012); and in cultivated *Agave* species (from 21 to 66%; Eguiarte et al., 2013). Also, in the Mesoamerican common bean a ∼20% reduction in genetic variation has been reported (Schmutz et al., 2014). However, the amount of genetic diversity that is lost along domestication depends on several factors, including the severity and the number of bottlenecks, the strength of selection and human management (Gepts, 2014). To properly assess impact of domestication on the genetic diversity of *P. coccineus*, genomic data comparing wild and cultivated populations is necessary.

The use of genomic tools also allows to characterize diversity and differentiation patterns across genomes. Regions or variants that departure from neutral predictions are probably influenced by selective pressures and are tagged as candidates. Applying this approach to crop species and their wild relatives allows to distinguish loci affected during domestication, whereas comparisons between landraces and/or improved cultivars measure the effect of subsequent selection (Tang et al., 2010; Gepts, 2014). Furthermore, hypotheses about phenotypic convergence in crops can be tested. In other words, if the same genes or genomic regions were affected during the domestication process of different species.

Here, we aim to deal with the previous knowledge gaps by using genomic data to (1) provide information about the domestication history of *P. coccineus* and its current evolutionary dynamic in Mexico, in particular to analyze the occurrence of a single or multiple domestication events in Mexico; (2) examine the extent of the domestication bottleneck in this species by comparing the levels of genetic diversity and geographic patterns of the wild, feral and domesticated Mexican populations; and (3) identify candidate loci under natural and artificial selection in *P. coccineus* genome.

## Materials and Methods

### Plant Material and SNP Genotyping

*Phaseolus coccineus* individuals from 10 wild, three feral and 11 cultivated Mexican populations and one cultivar from Spain were analyzed, as well as plants from the breeding line Blanco Tlaxcala. Taxonomic and wild/feral/domesticated categories were assigned based on morphology and habitat observations. Only one of the wild populations that were sampled corresponds to subsp. *striatus*, the rest belong to subsp. *coccineus*. A population was classified as feral if it was growing out of cultivation and presented intermediate traits between wild and domesticated forms. The Mexican samples cover the species distribution and main cultivation areas at the national level. As outgroups, samples from the closely related species *P. vulgaris* (three wild and one cultivated) and *P. dumosus* (seven cultivated) were included (Supplementary Table S1). For the three species, the samples size of each population varied between three to 16 individuals.

Sampling was performed during September–December of 2014 and 2015. In the case of the wild populations, tissue from young leaves was collected and stored in silica until processed. Seeds from cultivars were collected and germinated at the Instituto de Ecología, UNAM. DNA was extracted using DNeasy Plant Mini Kit (Qiagen). DNA samples were genotyped at the Institute for Genomic Diversity at Cornell University (Services | Institute of Biotechnology, 2017). Sequencing libraries were constructed using enzymes PstI and BfaI following the Genotype by Sequencing (GBS) protocol of Elshire et al. (2011). A total of 326 samples were processed in four plates of ninety six samples each, multiplexed and sequenced on four lanes of Illumina HiSeq 2500 (100 bp, single-end reads).

Reads were aligned to *P. vulgaris* reference genome v1.0 (Phytozome) DOE-JGI and USDA-NIFA, http://phytozome.jgi.doe.gov/ (Phytozome, 2017) using bwa v 0.7.8-r455; (Li and Durbin, 2009). Demultiplexing, initial quality control, assembly and SNP discovery were made with TASSEL pipeline v3.0.174 (Glaubitz et al., 2014). Assembly and SNP discovery were performed independently for two sets of data, one containing samples from *P. vulgaris, P. dumosus*, and *P. coccineus* (VDC group), which are the domesticated species of the Vulgaris clade (Delgado-Salinas et al., 2006); and the other data set only including *P. coccineus* samples. SNPs were filtered in VCFtools 0.1.15 (Danecek et al., 2011) using the following parameters for the two data sets: (1) VDC group: maximum missingness threshold 20% per individual; minimum mean depth 10X; minimum allele frequency (MAF) 0.01; minimum allele count 90%; and only SNPs mapped in chromosomes. (2) *Phaseolus coccineus*: maximum missingness threshold 30% per individual; minimum mean depth 5X; MAF 0.02; minimum allele count 80%; and only SNPs mapped in chromosomes.

Filtered SNP data, species occurrence data and scripts used for the analyses are available at Dryad Repository under the identifier doi: 10.5061/dryad.q343c.

### Inferring Population Structure and Phylogenetic Relationships

We inferred the population structure of *P. coccineus* because different genetic clusters are expected to occur due to the isolation and environmental and cultural heterogeneity in which this species occurs. For this, the software Admixture v1.3 (Alexander et al., 2009) was used to infer population structure of *P. coccineus*. Values of *K* ranging from one to twenty were tested, and the value that exhibited the lowest cross-validation error was chosen. Then, we examined the phylogenetic relationships between the genetic groups, both cultivated and wild, and if each cluster forms a monophyletic clade. This phylogenetic analysis was also used as a preliminary approach to identify the plausible number of domestication events for the Mexican cultivated *P. coccineus* (see below for other analyses). Specifically we examined if the cultivated samples was recovered as a monophylogenetic group. For the phylogenetic analysis, wild and cultivated samples of *P. coccineus, P. vulgaris*, and *P. dumosus* were analyzed under three schemes:

First, a Maximum-Likelihood based approach was carried out with the FastTree software (Price et al., 2009). For this, a mix of Nearest-Neighbor Interchange and Subtree-Prune and Regraft moves (NNI+SPR) was considered for topology and branch-length optimization and the General-Time Reversible with a single rate per site model (GTR+CAT) was included as nucleotide substitution model. Because FastTree only considers those SNPs identified as fixed within individuals (i.e., homozygous), but polymorphic among individuals, only the 82% of the total VDC subset (41,223 SNPs) were considered in this analysis. Second, a phylogenetic network based on the Neighbor-net algorithm and Patristic Distances with GTR+I+G correction was estimated with SplitsTree (Huson and Bryant, 2006) software. Lastly, we employed a Bayesian multispecies coalescent model (Rannala and Yang, 2003) to estimate the phylogenetic relationships among well-supported clades within *P. coccineus* solely. We used the program SNAPP 1.3.0 (Bryant et al., 2012), included in the package BEAST 2.4.5 (Bouckaert et al., 2014) to infer species trees directly from biallelic genetic data. We used the eight main genetic clusters (see section Results) inferred by Admixture as *a priori* designated species and the Wild-TMVB cluster was partitioned in two, taking into account the ML topology of that cluster. Because SNAPP does not incorporate missing data, we selected a subset of our taxonomic sampling that maximized the number of SNPs available. The final analysis retained a total of 600 SNPs under linkage equilibrium; without any missing data and considering a minimum of five individuals from each cluster of the designated species. We used SNAPP’s default settings and ran the analysis for 1,000,000 generations sampling every 1,000 generations. We evaluated the convergence (i.e., short variation in -lnL scores, ESS > 100) from our runs by examining log files with the program Tracer 1.5 (Drummond and Rambaut, 2007). We analyzed the tree files with SNAPP-TreeSetAnalyser 2.4.5, to identify species trees that were contained in the 95% highest posterior density (HPD) set and using 10% of topologies as burn-in. Resulted tree files (cloudgrams) were visualized using DensiTree (Bouckaert, 2010).

### Population Genetics Statistics

To evaluate the existence and degree of the domestication bottleneck on *P. coccineus* we estimated genetic diversity and differentiation indices of the genetic groups inferred by the Admixture analysis (see section Results). Specifically, we used the Hierfstat package (Goudet, 2005) in R (R Core Team, 2017) to estimate per site heterozygosity and *F*_IS_, as well as pairwise *F*_ST_ among groups, performing a bootstrap (1,000) to obtain confidence intervals. To test the hypothesis that *n*_i_ = *n*_j_ (where *n*_i_ is the number of loci of the cluster *i* where *H*_Ei_ > *H*_Ej_, and *n*_2_ is the number of loci of the cluster *j* where *H*_Ej_ > *H*_Ei_) we used a pairwise χ^2^ tests with Bonferroni correction to avoid false positive results (Sokal and Rohlf, 1995). Also, we estimated the heterozygosity and *F*_IS_ at the sampling location (*P. coccineus* dataset) and at the species level (VDC dataset) applying the same test.

### Multiple vs. Single Domestication Events Test

In order to confirm the hypothesis of a single domestication event in Mexico suggested by our phylogenetic analyses (see section Results) we applied the Approximate Bayesian computation (ABC; Beaumont et al., 2002) method implemented in DIYABC 2.04 (Cornuet et al., 2014). Preliminary tests included comparisons among three scenarios with 3 × 10^6^ simulated datasets (1 × 10^6^ each scenario) in which the position of the Wild-Sierra Madre Occidental (Wild-SMOCC) clade was evaluated (see section Results, Supplementary Figure S1). Our final estimation included 4 × 10^6^ simulated datasets (2 × 10^6^ each scenario) considering the Wild-SMOCC population fixed as sister clade of the Wild-Trans-Mexican Volcanic Belt (Wild-TMVB) populations (see section Results). The number of domestication events was tested as follows: multiple events (Scenario 1, Supplementary Figure S2) vs. a single one (Scenario 2, Supplementary Figure S2). The DIYABC approach was also applied to estimate the time at which domestication occurred, as well as other demographic parameters such as effective population size (*Ne*). A subsample from the SNAPP dataset (279 SNPs) and the scheme of eight clusters were used to set populations in DIYABC (**Figure 2B**). Priors were set as follow: log-uniform distributions across all parameters, *Ne* ranging from 100 to 100,000 individuals, mutation rate set to 10^-8^–10^-6^ across SNPs, and divergence times among populations set to 10–100,000 generations ago (**Table 1**).

**Table 1:** Estimations of effective population sizes of the best-fit DIYABC model (single domestication) for *Phaseolus coccineus* in Mexico.

Genetic group	Minimum prior value	Maximum prior value	Average posterior value	95%CI
Wild-SUR-CH	100	1 × 10^5^	1.0 × 10^5^	9.98 × 10^4^–1 × 10^5^
Wild-SMOCC	100	1 × 10^5^	8.94 × 10^4^	8.01 × 10^4^–9.5 × 10^4^
Wild-TMVB	100	1 × 10^5^	8.94 × 10^4^	8.01 × 10^4^–9.5 × 10^4^
Wild-striatus	100	1 × 10^5^	9.68 × 10^4^	8.9 × 10^4^–1 × 10^5^
Cult-OV	100	1 × 10^5^	8.83 × 10^4^	7.5 × 10^4^–1 × 10^4^
Cult-SUR-CH	100	1 × 10^5^	6.39 × 10^4^	5.7 × 10^4^–9.38 × 10^4^
Cult-TMVB	100	1 × 10^5^	9.36 × 10^3^	8.39 × 10^3^–1.54 × 10^4^
Cult-SMOCC	100	1 × 10^5^	8.57 × 10^3^	6.01 × 10^3^–1.2 × 10^4^

*Please refer to text to understand what acronyms stands for.*

We compared the fit of the single vs. multiple domestication events scenarios by estimating their posterior probabilities: with the obtained reference tables from each scenario, we ranked the simulated datasets in order of increasing distance to the observed data considering direct and logistic approaches (Beaumont et al., 2002; Cornuet et al., 2014). Distance between datasets was based on summary statistics, estimated from the empirical and simulated sets. We performed a pre-evaluation step using a principal components analysis (PCA), to ensure that at least one (or more) scenarios would produce simulated datasets close enough to the empirical data. The PCA was based on a set of 5,000 simulated datasets, generated from the parameters’ prior distributions (Supplementary Figure S3).

### Identifying Candidate Loci

We used the wild and cultivated samples of *P. coccineus* to identify candidate loci related to domestication, to cultivar diversification, and to natural selection. Before the candidate SNPs analysis, an additional filter based on linkage disequilibrium (LD) was applied. To determine the threshold distance at which there is no LD, we estimate the inter-variant allele correlations (*r*^2^) using PLINK 1.9 (Chang et al., 2015). To distinguish LD due to physical distance (bp), the *r*^2^ was estimated for SNPs located in the same and in different chromosomes. The distance threshold was established in 3,000 bp, so that SNPs closer than this distance were removed.

This LD-filtered dataset was analyzed with two different approaches for outlier detection: the R package pcadapt (Luu et al., 2017) and BayeScan 2.1 (Foll and Gaggiotti, 2008). Only loci identified by the pcadapt and BayeScan methods were considered as candidate loci. Pcadapt detects candidate SNPs assuming that these are outliers with respect to how they are related to population structure. By contrast to population-based approaches, pcadapt does not require grouping individuals into populations and handles admixed individuals (Luu et al., 2017). BayeScan instead uses differences in allele frequencies of pre-defined populations, in this case the genetic clusters previously established by Admixture.

In both approaches, three separate analyses were performed with each method to detect signatures of different types of selective pressure. First, to detect candidate domestication loci, wild and cultivated samples of *P. coccineus* were included, and feral individuals were removed. In this case, for the pcadapt analysis, only the first principal component was assessed because it explains the difference between wild and cultivated populations (see section Results). Also, an additional SNPs filter was made and MAF were adjusted to consider SNPs present in at least five individuals. For this dataset, that is MAF = 0.023. For Bayescan no additional filter was made. Second, to identify loci related to diversification in the context of domestication, only cultivated samples were analyzed. In the pcadapt analyses, the first six components were assessed because they explain the genetic structure of populations, and MAF threshold was set to 0.038 to excluded alleles present in less than five individuals. Notice that in this case, diversification refers to the phase that follows initial domestication and involves the spread and adaptation to different agro-ecological and socio-cultural environments (Meyer and Purugganan, 2013). Lastly, to detect natural selection signatures, we focused both methods on wild samples. Again, for the pcadapt analyses the first six components were assessed and the MAF threshold was set 0.055 to exclude SNPs present in less than five individuals. In all cases, no additional filter was made for BayeScan.

The false discovery rate threshold applied in pcadapt and BayeScan were 0.005 and 0.05, respectively. To compare how genetic variance is explained by candidate SNPs and by data set LD filtered, PCAs were made using the SNPrelate package (Zheng et al., 2012).

Using Phytozome’s JBrowser, the putative function and tissue of expression of these loci was examined by looking for the annotation of the selected SNPs in *P. vulgaris* genome v 2.1 (DOE-JGI and USDA-NIFA^1^). For each annotated loci we looked for homologous proteins with the highest similarity in other plants, and examined if the homolog genes in *Glycine max* (soybean) were among the domestication-related loci associated with flowering time and seed size in this species (Zhou et al., 2015).

## Results

### Sampling and SNP Genotyping

**FIGURE 1:**
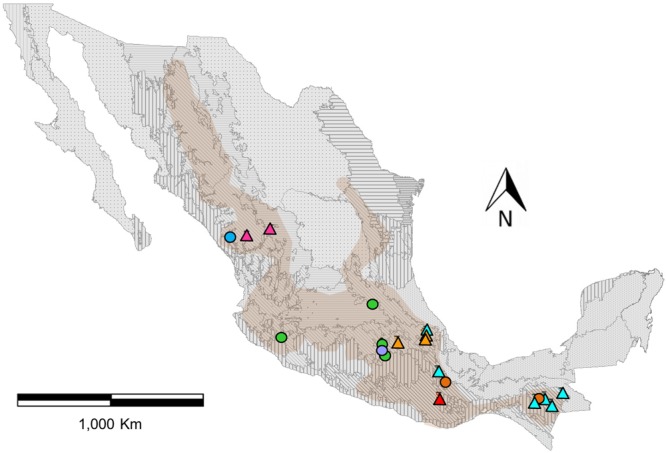
Distribution map of genotyped populations of *Phaseolus coccineus* in Mexico, assigned to eight genetic clusters with the program Admixture (colors). Circles indicate wild populations and triangles show cultivated populations (**Figure 2**). Frames indicate the seven first-level ecoregions (9–15) present in Mexico (level 1) and boundaries represent 21 second-level nested ecoregions. Shaded area shown the potential geographical distribution of *P. coccineus* in Mexico (López-Soto et al., 2005).

A total of 296 individuals representing four ecoregions of Mexico (as defined in Instituto Nacional de Estadística, Geografía e Informática (INEGI), Comisión Nacional para el Conocimiento y Uso de la Biodiversidad (CONABIO), and Instituto Nacional de Ecología (INE), 2008) were sampled and successfully genotyped (**Figure 1**). After assembly and SNP discovery, the VDC group dataset contains 241 individuals of *P. coccineus*, 20 of *P. vulgaris* and 35 of *P. dumosus*, 50 273 SNPs, 2.24% mean missing data per individual, and a mean depth per site of 58.63. The *P. coccineus* dataset includes 242 individuals (91 wild; 20 feral; 131 cultivated), 42,548 SNPs, 3.97% mean missing data per individual, and a mean depth per site of 50.41.

### Inferring Population Structure and Phylogenetic Relationships

**FIGURE 2:**
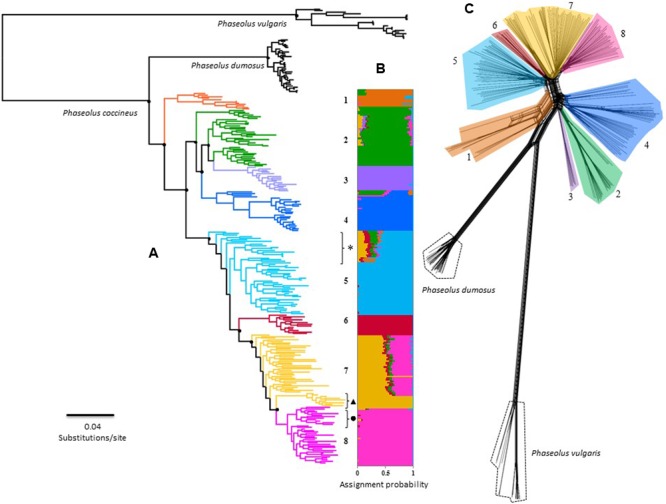
Overall phylogenetic relationship among 242 individuals of *P. coccineus* from Mexico. Numbers and colors represents the eight main genetic clusters solved by Admixture: (1) Wild-SUR-CH, (2) Wild-TMVB, (3) Wild-striatus, (4) Wild-SMOCC, (5) Cult-SUR-CH, (6) Cult-OV, (7) Cult-TMVB and (8) Cult-SMOCC. Please refer to text to understand what acronyms stands for. **(A)** Maximum-Likelihood tree, main doted clades indicated support bootstrap values >75%. Black dot next to cluster 8 indicates the Blanco Tlaxcala breeding line; dark triangle shows the Spanish population; feral samples are indicated with an asterisk. **(B)** Individual assignment based on 42,548 SNP’s solved with Admixture. **(C)** Rooted Neighbor-Net topology achieved by SplitsTree.

The *K-*value that presents the lower error rate in Admixture analysis was eight (Supplementary Figure S4). Half of the genetic groups correspond to the cultivars from the Trans-Mexican Volcanic Belt (Cult-TMVB), Sierra Madre del Sur and Chiapas Highlands (Cult-SUR-CH), Sierra Madre Occidental (Cult-SMOCC) and Oaxaca Valley (Cult-OV). The other half of the genetic clusters belong to wild populations from the Trans-Mexican Volcanic Belt (Wild-TMVB), Sierra Madre del Sur and Chiapas Highlands (Wild-SUR-CH), Sierra Madre Occidental (Wild-SMOCC) and subsp. *striatus* population, located in the TMVB (Wild-*striatus*; **Figure 2**). The genetic clusters seem to be related to geographic distances (**Figure 1**), except the population Wild-*striatus*, which is geographically close to populations of *P. coccineus* subsp. *coccineus* but seems genetically isolated. Samples from the Spanish population (**Figure 2B**, triangle) were assigned to the Cult-TMVB genetic group, but unlike the individuals of this cluster, samples from Spain do not present a mixed ancestry. Regarding samples of the breeding line Blanco Tlaxcala (**Figure 2B**, circle), they are grouped with landraces from Cult-SMOCC cluster.

The phylogenetic hypotheses constructed with FastTree and SplitsTree (**Figures 2A,C**) are consistent with the Admixture genetic groups (**Figure 2B**). Nevertheless, both analysis suggested the Wild-TMVB group as a paraphyletic clade. ML topology revealed a finer-scale structure, identifying three paraphyletic clades within this genetic cluster, and Wild-*striatus* cluster is a nested clade differentiated from the rest of the Wild-TMVB group (**Figure 2**). Remarkably, the domesticated populations integrate a monophyletic clade statistically well supported, suggesting a unique domestication event for the Mexican populations. Nevertheless, these phylogenetic hypotheses do not allow to distinguish the genetic pool from which domestication took place, although the Wild-SUR-CH genetic cluster can be discarded.

The ML and Neighbor-Net topologies in which *P. dumosus* and *P. vulgaris* were included, positioned *P. dumosus* as a sister group of *P. coccineus* (**Figure 2A**). However, the SplitsTree method indicated a basal reticulate pattern among *P. dumosus, P. coccineus*, and *P. vulgaris* (**Figure 2C**), suggesting ancestral gene flow, but not recent. Furthermore, there is no evidence of recent gene flow between wild and cultivated groups, but only within genetic clusters (**Figure 2C**).

**FIGURE 3:**
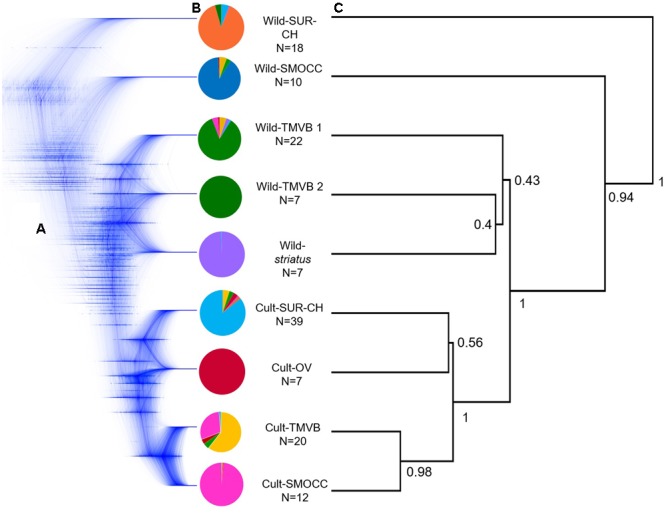
**(A)** Cloudgram depicting topologies of 9,999 species trees obtained from an analysis of 600 single nucleotide polymorphism loci from 124 *P. coccineus* using SNAPP; **(B)** average assignment probability achieved by Admixture of selected individuals considered in species tree analyses based on nine groups; **(C)** associated root canal depicting a consensus topology from SNAPP analysis. Nodal support values on the root canal are posterior probabilities that correspond to strongly supported nodes designated *a priori* in the species tree analysis.

Regarding SNAPP cloudgram (**Figures 3B,C**), 53 single topologies summarize the 95% HPD consensus tree, indicating a different divergence pattern in which Wild-TMVB populations are the closest clade to the domesticated group. Nevertheless, the complex assignment of individuals within Wild-TMVB and Wild-*striatus* are shown in a non-solved pattern within the cloudgram as well as in low values of nodal support in the consensus topology (**Figure 3C**). Despite these main inconsistencies between ML and Neighbor-Net vs. SNAPP topologies, all hypotheses favor the occurrence of a single domestication event.

**FIGURE 4:**
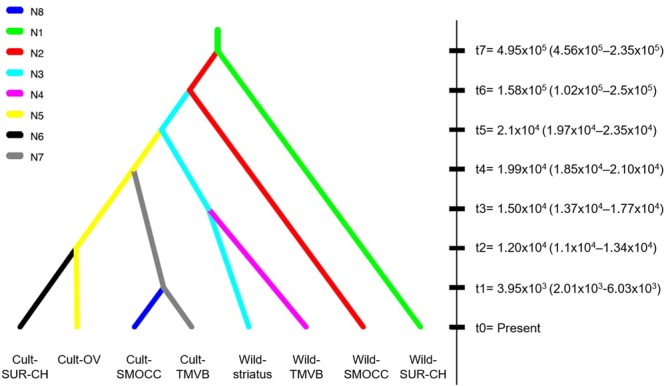
Best-fitted domestication scenario of *P. coccineus* achieved with DIYABC. Split times in generations (tn) indicated the average posterior value estimated after Bayesian Computations (95% CI).

In regards of the ABC-based computations, the model comparisons in preliminary trials indicated scenarios where the Wild-SMOCC population that are paraphyletic to Wild-TMVB yielded a higher probability in both direct and logistic approaches (Supplementary Figures S1, S2). A final test indicated that the most likely scenario was a single domestication event, being the Wild-TMVB group the closest to the domesticated clade (**Figure 4**; Scenario 2, direct *P* = 0.786, logistic *P* = 1.0), which is congruent with the results of SNAPP phylogenetic analyses. Evaluation of the posterior predictions via PCA indicated that parameter values and summary statistics from the simulated datasets based on Scenario 1 closely matched the empirical data (Supplementary Figure S3).

### Wild and Domesticated Population Genetics Statistics

**FIGURE 5:**
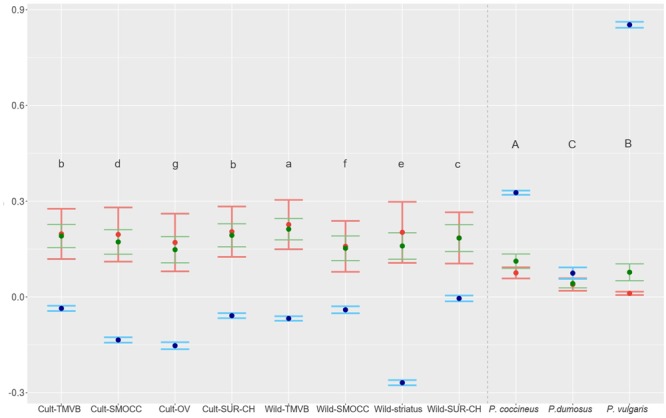
*H*_E_ in green (variance interval), *H*_O_ in red (variance interval) and inbreeding coefficient in blue (95% IC) estimated for genetic clusters of *P. coccineus* and for species of VDC group. Letters show groups that are statistically different and are decreasingly ordered according *H*_E_.

High levels of genetic diversity were found in wild and cultivated populations (**Figure 5**). At the genetic cluster level, the Wild-TMVB group presented the highest diversity and the Cult-OV group the lowest. No clear pattern in the amount of diversity was observed between wild and cultivated clusters. There were cultivated groups with high genetic variance (Cult-SUR-CH and Cult-TMVB), and wild clusters that presented lower diversity than cultivated populations (Wild-SMOCC). At the location level (Supplementary Table S2), the samples from Spain (*H*_E_ = 0.134) and Oaxaca Valley (*H*_E_ = 0.148) presented the lowest diversity, and the highest was found in wild population located in Tlalpan, Mexico City (*H*_E_ = 0.208). Regarding species, *P. coccineus* showed the highest diversity and *P. dumosus* the lowest.

**FIGURE 6:**
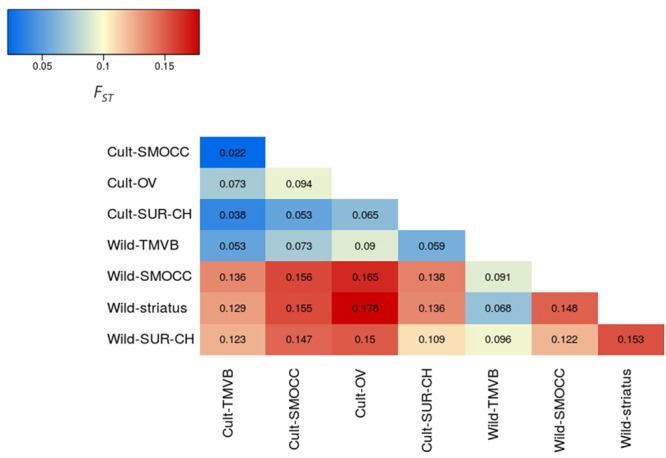
Heatmap representing the pair *F*_ST_ values between genetic clusters.

Outstandingly, *H*_O_ was greater than *H*_E_ in all the genetic groups except in the Wild-SUR-CH cluster, resulting in negative values of *F*_IS_. Within the groups with an excess of observed heterozygosity, Wild-*striatus* had the lowest inbreeding coefficient (**Figure 5**). On the contrary, at the species level *P. vulgaris* showed a deficit of heterozygotes, showing a high *F*_IS_. The inbreeding coefficient is positive when estimated taking into account all *P. coccineus* samples. This is caused by the Wahlund effect, which is the reduction of heterozygosity due to subpopulation structure. Regarding pairwise differentiation index, *F*_ST_ values ranged from 0.022 (Cult-TMVB vs. Cult-SMOCC) to 0.178 (Cult-OV vs. Wild-*striatus*; **Figure 6**). As expected, the pair *F*_ST_ values are greater between wild genetic groups than between cultivated genetic clusters (**Figure 6**).

Cultivated populations of *P. coccineus* show smaller effective population sizes than wild populations. In some cases, like in Cult-TMVB and Cult-SMOCC, *Ne* was one order of magnitude smaller than in the rest of the populations. On the contrary, the genetic cluster Wild-SUR-CH had the biggest *Ne* (**Table 1**). The most recent split was estimated to happen 3.9 × 10^3^ generations ago, and occurred between the Cult-SMOCC and the Cult-TMVB clusters. On the contrary, the oldest split event was dated in 4.95 × 10^5^ generations ago between the Wild-SUR-CH and the rest of *P. coccineus* clade. The split event that separates wild and domesticated samples was dated about 2.1 × 10^4^ generations ago (**Figure 4**). Since *P. coccineus* is usually treated as an annual when cultivated, that represents 21,000 years ago. In the case of wild, perennial plants, one generation could be more than a year.

### Identifying Candidate Loci

Before LD filtering, the mean *r*^2^ value among SNPs located in the same chromosome separated by a maximum distance of 10,000 bp was 0.151. After eliminating SNPs closer than 3,000 bp, the mean *r*^2^ was 0.063 (Supplementary Figure S5). In the case of SNPs from different chromosomes, the mean *r*^2^ was 0.022. This low LD is not due to the closeness, but rather by factors like populations structure. Interestingly, the pattern in the decay of LD differed between genetic groups, with the fastest decay and lowest *r*^2^ in cultivated and wild populations from the TMVB. Meanwhile, Wild-*striatus*, Wild-SURCH and Cult-OV had the slowest LD decay and highest *r*^2^ values (Supplementary Figure S5). After filtering, the data set for candidate loci contained 11,693 SNPs distributed across the 11 chromosomes. In the central region of most of the chromosomes, there is a reduction in SNP density, probably due to centromeres (Supplementary Figure S6).

Using the pcadapt package, 47 SNPs were identified as candidate domestication loci; 342 involved in cultivar diversification; and 1,030 potentially under natural selection. Despite the great number of candidate SNPs that were identified, few are shared among selection types (Supplementary Figure S7). In the case of the BayeScan analyses, 469 candidate SNPs for domestication were identified; 16 related to cultivar diversification; and 12 candidates associated with natural selection. None of these SNPs were shared among the three BayeScan analysis.

**FIGURE 7:**
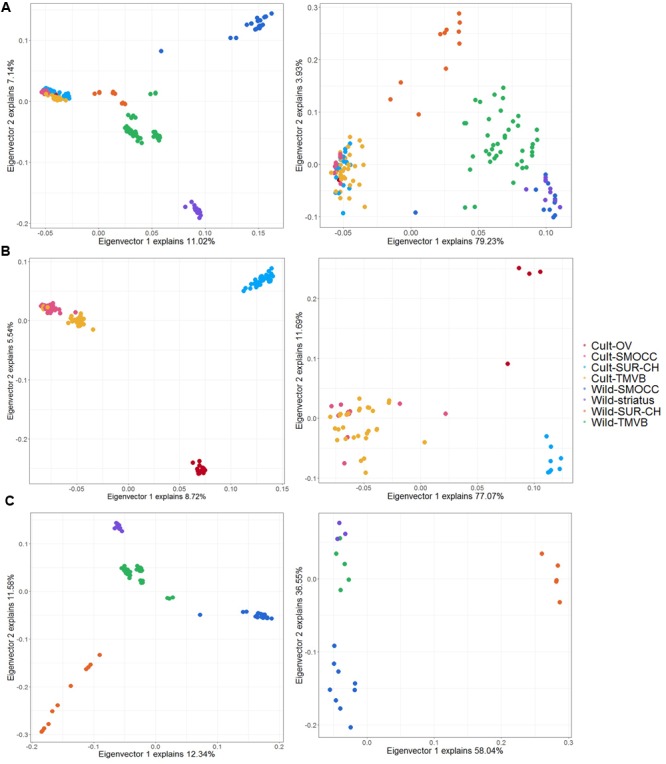
Principal components analysis plot for the first two principal components using LD dataset (left) and candidate SNPs identify in both in PCAdapt and BayeScan analysis (right). **(A)** Analysis including cultivated and wild samples (no feral) to detect domestication-related SNPs. **(B)** Analysis of cultivated populations to distinguish cultivar diversification-related loci. **(C)** Analysis of wild samples to detect signatures of natural selection.

Twenty four SNPs related to domestication, 13 to cultivar diversification and eight to natural selection were detected by both approaches and considered as candidate loci for further analyses (Supplementary Table S3). The genetic variance explained by the candidate SNPs compared to the 11,693 SNPs used previously changed dramatically (**Figure 7**). Notably, the genetic and geographic structure of wild and cultivated groups can be recovered by these few candidate SNPs (**Figures 7B,C**) and a clear separation of wild and domesticated populations is observed (**Figure 7A**).

Four SNPs of the candidate domestication loci were found to be annotated in *P. vulgaris* genome, one of the candidate loci under natural selection and none of the candidate loci for cultivar diversification (Supplementary Table S3). Three of the annotated candidate domestication loci (Phvul.001G232200, Phvul.007G256000, Phvul.009G156400) are highly expressed in flowers, flower buds or young pods, and the remaining locus (Phvul.002G145600) is highly expressed in green mature pods. All these loci have their highest similarity homologs in *G. max* genome v2.0 (Schmutz et al., 2010), but none of these correspond to the domestication-related loci previously identified by Zhou et al. (2015). The annotated candidate locus for natural selection (Phvul.003G197500) is highly expressed in roots and steam and corresponds to a calmodulin binding protein-like, which also has an homolog in *G. max.*

## Discussion

### A Single Domestication Event for Mexican *P. coccineus* in the TMVB

Spataro et al. (2011) and Rodriguez et al. (2013), using SSR data, suggested two domestications events of *P. coccineus*, one in Mexico and the other in Guatemala-Honduras. The genomic data generated in this work indicates a unique domestication event for the cultivated populations from Mexico (**Figures 2, 3**). This includes Chiapas populations (Cult-SUR-CH), which are geographically and culturally closer to Guatemala than to Central and Northern Mexico. However, no samples from Guatemala and Honduras were included, therefore a second domestication event in this area cannot be discarded with the present data. Nonetheless, based on the results from SNAPP and DIYABC analyses, we were able to identify Wild-TMVB as the genetic pool from which domestication started in Mexico (**Figure 3**).

The most recent divergence time, that corresponds to the separation between cultivated groups of SMOCC and TMVB, was dated in 3,950 generations ago (**Figure 4**, t1). Assuming one generation per year in cultivated populations, this represents 3,950 years. But divergence between the cultivated and wild clades was dated in 21,000 generations (**Figure 4**, t5). This date is out of range of any plant domestication event and it seems unlikely. There are evolutionary processes that may affect these estimations. Processes like selection, population subdivision and incomplete lineage sorting may result in an overestimations of divergence times because increase the time to coalescence, that is, the time it takes for the two sequences to find their common ancestor (Albrechtsen et al., 2010; Angelis and Dos Reis, 2015). In *P. coccineus*, the selection made by humans during domestication and the high population structure in wild and domesticated groups probably has resulted in overestimated divergence times. Also, it has to be considered that wild populations are perennial and thus generation times may be longer than a year.

The genetic findings suggest that *P. coccineus* domestication likely occurred from TMVB’s material, pinpointing the domestication of this species to a particular region within the large Mexican territory where it is cultivated nowadays. Other sources of information could be incorporated to confirm this, using our findings as a geographic reference. If confirmed, identifying the TMVB as the area where domestication started for this species is interesting and important from an evolutionary, cultural and conservation perspective. The TMVB is the most recent mountainous region of Mexico, a biodiversity hotspot and it has a complex bio- and phylogeographic history characterized by following a sky-island dynamic during the last 2 Myr (Mastretta-Yanes et al., 2015). Culturally it became prominent during the Mexica Empire, and has been the most populated part of Mexico since little before the Spanish conquest (Bataillon, 1972). This has derived in other important cases of domestication to occur in this region. For instance, this central region was where the introgression of *Zea mays* ssp. *parviglumis* and *Z. mays* ssp. *mexicana* occurred during the domestication of maize (van Heerwaarden et al., 2011). However, human occupation in this area is also a concern for conservation, because the growth of urbanization and high-input agriculture in this area threat both *P. coccineus* landraces and wild populations (CONABIO and IUCN, 2016).

Besides genetic data, a Mexican domestication origin of *P. coccineus* is also supported by the several names that this bean has among different cultures. For instance, it is called tekómari in Chihuahua (Tarahumara indigenous language); tasukhu in Hidalgo and Puebla (Otomi); ayocote in central states of Mexico (Nahuatl); shaushana or xaxana in Veracruz (Totonaco); ma-má-ja (Mazateco) in Oaxaca; and botil or shbotil chenec in Chiapas (Tzeltal) (Salinas, 1988). Associated to these groups, there is also considerable traditional knowledge regarding the cultivation and use of *P. coccineus* species (e.g., Monroy and Quezada-Martínez, 2010).

### Historic and Recent Gene Flow among Wild, Feral and Domesticated Populations

The individuals identified as feral clustered in the domesticated clade (**Figure 2A**), suggesting that they are escaped cultivars. This questions the hypothesis of an hybrid origin between wild and cultivated populations (Salinas, 1988) and contrasts with previous studies of feral *P. vulgaris* populations in Mexico (Papa and Gepts, 2003), where weedy populations appear to be genetically intermediate between domesticated and wild populations, and not cultivar escapees. Interestingly, the three collected feral populations belonged to the same genetic cluster (Cult-SUR-CH) and presented high levels of mixed ancestry, of which only a small proportion corresponds to wild clusters (**Figure 2B**). Since little evidence of gene flow was found in SplitsTree (**Figure 2C**), probably the mixed ancestry is due to shared polymorphisms or ancestral gene flow, rather than recent introgression events.

The breeding line Blanco Tlaxcala grouped with SMOCC landraces. Probably, breeding practices have acted over specific regions rather than over all the genome. The individuals of this breeding line did not present mixed ancestry, despite that Blanco Tlaxcala was developed using a multi linear method (Vargas-Vázquez et al., 2012). This suggests that all lines used to generate Blanco Tlaxcala belonged to the same genetic cluster (Cult-SMOCC), and they were submitted to several rounds of strong selection, decreasing genetic variation.

Contrary to what was reported by Spataro et al. (2011) and Rodriguez et al. (2013), samples from Spain clustered within the TMVB landraces, indicating that this European population was originated by the introduction of individuals of the Cult-TMVB group into Spain. Nevertheless, because just one European population was analyzed, no general pattern can yet be inferred. Notably, Spanish samples did not present mixed ancestry, meanwhile the rest of the individuals of this genetic group did (**Figure 2B**). Probably the genetic bottleneck that originated European populations and the isolation from wild relatives and American landraces, have decreased the amount of shared ancestral polymorphisms between cultivars from TMVB and Spain.

It has been suggested that hybridization and introgression have played a major role in *P. coccineus* evolution, both in cultivated and wild populations (Escalante et al., 1994; Angioi et al., 2009; Spataro et al., 2011; Rodriguez et al., 2013). Our results showed mixed ancestry both in wild and cultivated clusters. However, little evidence of introgression and hybridization was detected, and mixed ancestry can also be due to shared ancestral polymorphisms. Nevertheless, wild and cultivated populations frequently coexist, therefore hybridization cannot be discarded and a formal test considering the number and size of introgressed regions and the direction of gene flow must be done.

### *Phaseolus coccineus* Is Highly Diverse and Structured

*Phaseolus coccineus* wild populations are divided in four genetic clusters that show considerable population differentiation. Similar levels of differentiation have been observed in several other highland species, which has been related to the high environmental variability and the complex geologic and climatic history of Mexico (Mastretta-Yanes et al., 2015). The extent of this differentiation in crop wild relative species has been mostly done with low resolution neutral makers (Bellon et al., 2009; Piñero et al., 2009) so it still needs to be further explored with genomic data. However, the present study and analyses in teosinte (van Heerwaarden et al., 2011; Aguirre-Liguori et al., 2017), highlight that there is high diversity contained in the genetic pools of crop wild relatives from Mexico.

Besides the diversity contained in wild relatives, one of the most important determinants in crop evolution is the level of genetic diversity contained in the domesticated populations, especially with reference to the wild ancestral gene pool. Genetic diversity reduction has been widely described in crop domestication (Hufford et al., 2013; Li et al., 2013; Schmutz et al., 2014; Renaut and Rieseberg, 2015). This reduction of genetic diversity is caused by genetic drift resulting from population bottlenecks, and by artificial selection (Gepts, 2014). This phenomenon was also described in *P. vulgaris* (Schmutz et al., 2014) but in *P. coccineus* no clear pattern of genetic reduction was found between the wild or cultivated genetic groups (**Figure 5**). The Wild-TMVB cluster presented the highest genetic variation, followed by the Cult-TMVB and Cult-SUR-CH groups. On the contrary, the Cult-OV and Wild-SMOCC clusters showed the lowest *H*_E_. Regarding effective population sizes, these were greater in wild than in cultivated genetic clusters, which is expected due to the genetic bottlenecks associated to domestication process. Nevertheless, *Ne* estimations of domesticated groups are in the order of 10^3^–10^4^. Taking together all results, these suggest that the genetic bottleneck during domestication was not severe. Other factors that may favor the maintenance of genetic diversity in *P. coccineus* are its high outcrossing rate (Escalante et al., 1994) and the fact that the genetic cluster from which domestication started (Wild-TMVB) presents the highest diversity. Little evidence of recent gene flow was detected, but early gene flow could also favor the amount of genetic diversity in cultivars.

Analyzing the genetic variance at the location level, Spanish samples presented the lowest diversity (Supplementary Table S2), which may be due to the recent demographic bottleneck that occurred during its introduction to Europe. Nevertheless, Oaxaca Valley also showed low genetic variation (Supplementary Table S2) and the ancestry analysis (**Figure 2B**) suggests that it has been genetically isolated from the other genetic clusters.

Regarding the inbreeding coefficient, the wild and cultivated genetic clusters presented negative *F*_IS_ values, indicating an excess of heterozygotes, except in the Wild-SUR-CH group. A possible explanation for this pattern is inbreeding depression, which effect in progeny has been studied in cultivars from Spain, finding that selfing affected germination, survival rate and seed weight (González et al., 2014). Also, a negative correlation was found between outcrossing rate and seed abortion in wild populations studied by Escalante et al. (1994). In the case of domesticated populations, the bottlenecks that they suffered during domestication may promote the accumulation of deleterious alleles and the increase of inbreeding depression, resulting in lower values of the inbreeding coefficient (Morrell et al., 2011). Opposite to what was expected, in *P. coccineus* the population with the lowest *F*_IS_ was a wild cluster (Wild-striatus). This population was previously studied by Búrquez and Sarukhán (1984), who found evidence of self-incompatibility, which is congruent with our results. A possible explanation for this pattern is the accumulation of deleterious alleles in the Wild-striatus cluster. Notably, no mixed ancestry was detected in this genetic group, indicating that it is genetically isolated from other populations despite being geographically close to other wild and cultivated TMVB populations. It is necessary to evaluate other populations of *P. coccineus* subsp. *striatus* to know if this is a common pattern and to explore the ecological and genetic causes and consequences of it.

### Adaptative Variation in Wild and Domesticated Populations

Mexico is an environmentally and culturally heterogeneous country, which favored crop genetic diversity. The distribution of *Phaseolus*, both cultivated and wild, involves an interaction with a wide range of different cultures, and isolated populations are exposed to diverse environmental conditions. For example, compared to *P. vulgaris, P. coccineus* grows in more humid environments, at cooler temperatures and at higher altitudes. Nevertheless, there are few studies that aim to elucidate the genetic basis of adaptation, especially for the wild populations of *Phaseolus* crop species (Bitocchi et al., 2017). Our outlier analyses listed some candidate SNPs that could be under artificial selection during the domestication and diversification stages, and others that could be under natural selection. Although most of these outliers are still not annotated, they could serve as a base for identifying population differentiation in adaptive variation, which is a needed step for genetic resources and crop wild relatives conservation (Maxted et al., 2012). Our study is based on GBS data, so *P. coccineus* genome is not fully saturated, and likely there are loci under selection that we did not sample. Nevertheless, this set of outliers are a first approximation to identify candidate loci to domestication and natural selection in runner bean.

The fact that no loci overlapped between domestication, diversification and natural selection categories shows that different selective processes were detected. This is to be expected because, in general, loci under natural selection and artificial selection related to domestication and diversification are expected to differ across the genome (Meyer and Purugganan, 2013).

The loci involved in domestication are expected to be specially related to the phenotypic changes of the domestication syndrome (Koinange et al., 1996), that is modifications in morphological and physiological traits like seed dispersal, seed dormancy, gigantism, increased harvest index and flowering time (Hammer, 1984). Most of the domestication-related loci identified here are still of unknown function, but the four that are annotated are highly expressed in flowers or pods (Supplementary Table S3). This is interesting because in the soybean, another legume, several domestication-related loci associated with flowering time have been identified (Zhou et al., 2015). However, no overlap among those loci and the ones identified here was found.

## Conclusion

The SNPs generated in this work provided high resolution data to understand the domestication of *P. coccineus.* Results suggest one domestication event for Mexico, which started from the wild genetic pool from TMVB. Furthermore, wild and domesticated populations are highly diverse and presented high values of *Ne*, suggesting that the demographic bottleneck due to domestication was not severe. These genomic analyses allow to highlight how the genetic signatures of domestication can be substantially different even between species of the same genus domesticated in the same geographic area. Common bean and scarlet runner bean are closely related species, nevertheless their reproductive strategies and domestication histories seem to be different: *P. vulgaris* tends to self-crossing, which theoretically facilities the domestication process, and it also suffered a severe domestication bottleneck. On the contrary, *P. coccineus* is an open pollinated species that presents high levels of genetic diversity and population structure, and its domestication did not result in a strong demographic bottleneck.

Our findings also show that both wild and domesticated populations of *P. coccineus* are highly structured. Most of the genetic clusters presented an heterozygotes excess, showing evidence of inbreeding depression. Interestingly, the population identified as *P. coccineus* subsp. *striatus* shows the greatest excess of heterozygotes and seems to be genetically isolated from other wild and cultivated populations. Contrasting with previous studies, our data shows that gene flow within and between wild and cultivated populations is not a common process. Fully testing this represents an area where further research is needed.

The levels of diversity and population differentiation found here support that the runner bean is a potential source of variability for several traits for plant breeding (Schwember et al., 2017). The data presented here highlights that for a better characterization of *P. coccineus* wild and cultivated forms there is still a need of more sampling, specially including Central American populations. Complete and annotated genomes of *Phaseolus* and other legume crops will facilitate not only comparative genomics, but will give a better knowledge of the evolution and domestication of this group of plants that has been independently domesticated by several human groups across its distribution.

## Author Contributions

AG-G, DP, and AD-S designed the study. AG-G made the molecular procedures. AG-G, AM-Y, and MS-A conducted the analyses. All authors revised the results and wrote the manuscript.

## Conflict of Interest Statement

The authors declare that the research was conducted in the absence of any commercial or financial relationships that could be construed as a potential conflict of interest. The handling Editor declared a shared affiliation and past co-authorship, though no other collaboration, with the authors and states that the process nevertheless met the standards of a fair and objective review.

## Footnotes

1 http://phytozome.jgi.doe.gov/
