# Supplementary material for: Domestication Genomics of the Open-Pollinated Scarlet Runner Bean (Phaseolus coccineus L.)
Source: Front Plant Sci. 2017 Nov 15;8:1891. doi: 10.3389/fpls.2017.01891 (PMC5694824; doi:10.3389/fpls.2017.01891)
Supplement: Supplementary file 1 [file Data_Sheet_1.pdf]

Supplementary Material

Domestication Genomics of the open-pollinated Scarlet Runner Bean (*Phaseolus coccineus* L.)

Azalea Guerra-García, Marco Suárez-Atilano, Alicia Mastretta-Yanes, Alfonso Delgado-Salinas, Daniel Piñero

*** Correspondence:** Azalea Guerra-García: azalea.guerra@iecologia.unam.mx

Table S1. Sampling information

Species	Status	State and location	Latitude	Longitude	Altitude
P. coccineus subsp. *coccineus*	Cultivated	Oaxaca, Cuilapam	16.99	-96.78	1577
P. coccineus subsp. *coccineus*	Cultivated	Durango	NA	NA	NA
P. coccineus subsp. *coccineus*	Cultivated	Veracruz, Frijol Colorado	19.59	-97.35	2419
P. coccineus subsp. *coccineus*	Cultivated	Chiapas, González León	16.51	-92.06	1579
P. coccineus subsp. *coccineus*	Cultivated	España	NA	NA	NA
P. coccineus subsp. *coccineus*	Cultivated	Chiapas, Nahá	16.94	-91.59	917
P. coccineus subsp. *coccineus*	Cultivated	Veracruz, Orilla del Monte	19.66	-97.29	2402
P. coccineus subsp. *coccineus*	Cultivated	Chaipas, Oxchuc	16.80	-92.32	2001
P. coccineus subsp. *coccineus*	Cultivated	Puebla, Tlalancaneca	19.36	-98.51	2372
P. coccineus subsp. *coccineus*	Cultivated	Durango, Regocijo	23.68	-105.12	2566
P. coccineus subsp. *coccineus*	Cultivated	Durango, Villa Unión	23.97	-104.04	1901
P. coccineus subsp. *coccineus*	Cultivated	Puebla	NA	NA	NA
P. coccineus subsp. *coccineus*	Feral	Veracruz, Altotonga	19.75	-97.25	1959

P. coccineus subsp. *coccineus*	Feral	Oaxaca, Huautla	18.10	-96.83	1746
P. coccineus subsp. *coccineus*	Feral	Chiapas, Zicanantán	16.75	-92.73	2408
P. coccineus subsp. *coccineus*	Breeding line	Blanco Tlaxcala	NA	NA	NA
P. coccineus subsp. *coccineus*	Wild	Jalisco, Ciudad Gúzman	19.58	-103.53	2100
P. coccineus subsp. *coccineus*	Wild	Oaxaca, Comaltepec	17.55	-96.53	2876
P. coccineus subsp. *coccineus*	Wild	Durango, Espinazo del Diablo	23.64	-105.82	1422
P. coccineus subsp. *coccineus*	Wild	Durango, Regocijo	23.68	-105.12	2566
P. coccineus subsp. *coccineus*	Wild	Ciudad de México, REPSA	19.32	-99.20	2328
P. coccineus subsp. *coccineus*	Wild	Querétaro, San Joaquín	20.93	-99.56	2381
P. coccineus subsp. *coccineus*	Wild	Chiapas, San Cristóbal	16.70	-92.60	2229
P. coccineus subsp. *coccineus*	Wild	Morelos, Tepoztlán	19.00	-99.13	1953
P. coccineus subsp. *coccineus*	Wild	Ciudad de México, Tlalpan	19.29	-99.19	2334
P. coccineus subsp. *striatus*	Wild	Morelos, Tres Marías	19.10	-99.21	3024
P. dumosus	Cultivated	Veracruz, Altotonga	19.75	-97.25	1959
P. dumosus	Cultivated	Chiapas, Chiquinivalvo	16.71	-92.86	1467
P. dumosus	Cultivated	Puebla, Cuetzalan	19.99	-97.54	1684
P. dumosus	Cultivated	Oaxaca, Huautla	18.10	-96.83	1746
P. dumosus	Cultivated	Chiapas, Motozintla	15.43	-92.33	2671
P. dumosus	Cultivated	Chiapas, Talquián	15.09	-92.08	1728
P. vulgaris	Cultivated	España	NA	NA	NA
P. vulgaris	Wild	Jalisco	NA	NA	NA
P. vulgaris	Wild	Ciudad de México, REPSA	19.32	-99.20	2328
P. vulgaris	Wild	Morelos, Yautepec	18.95	-99.08	1386

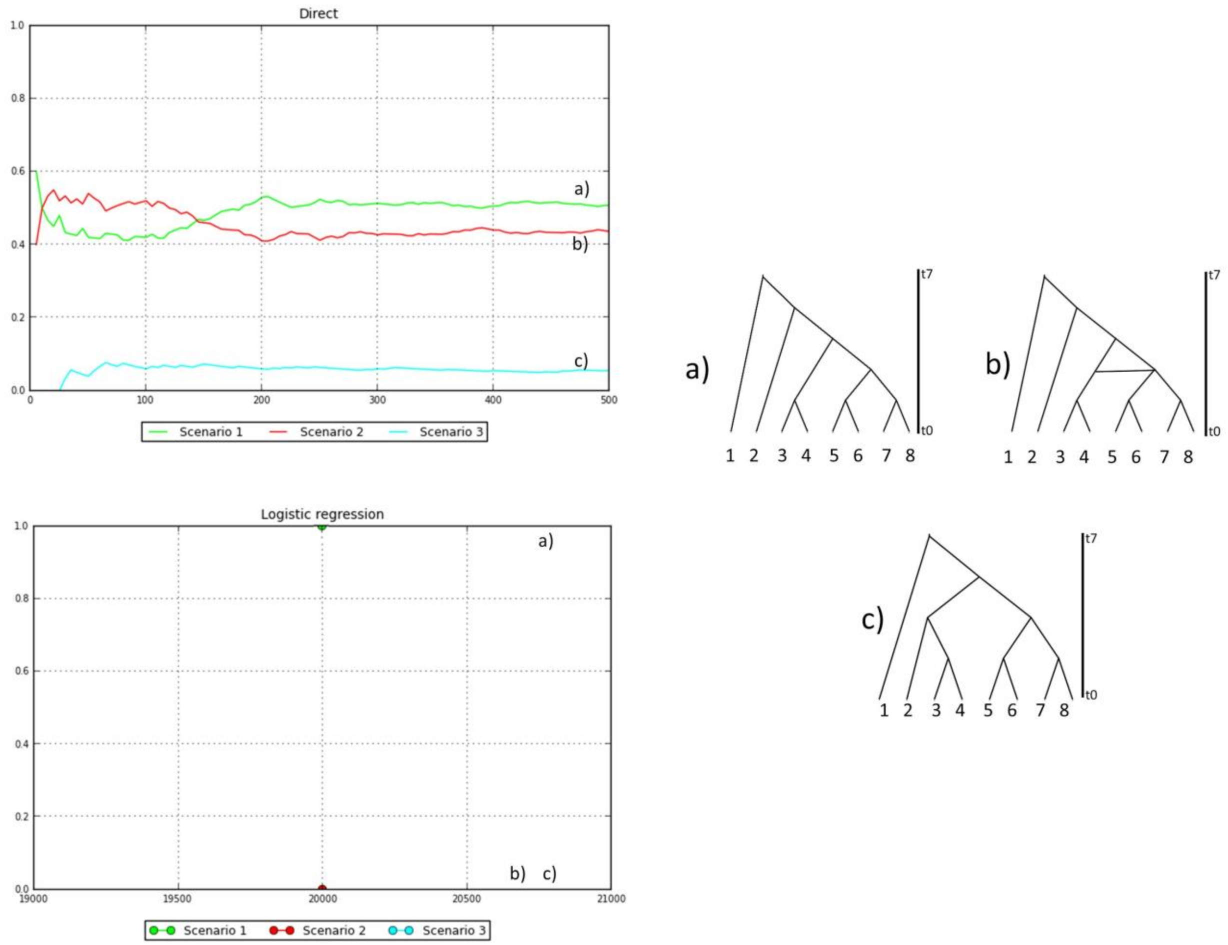

Figure S1. Posterior probabilities (Direct and Logistic) of the three main domestication scenarios tested with DIYABC, among eight wild and cultivated populations of Phaseolus coccineus in Mexico. In this preliminary evaluation: a) monophyletic origin of Wild populations b) and c) paraphyletic relationships among Wild populations.

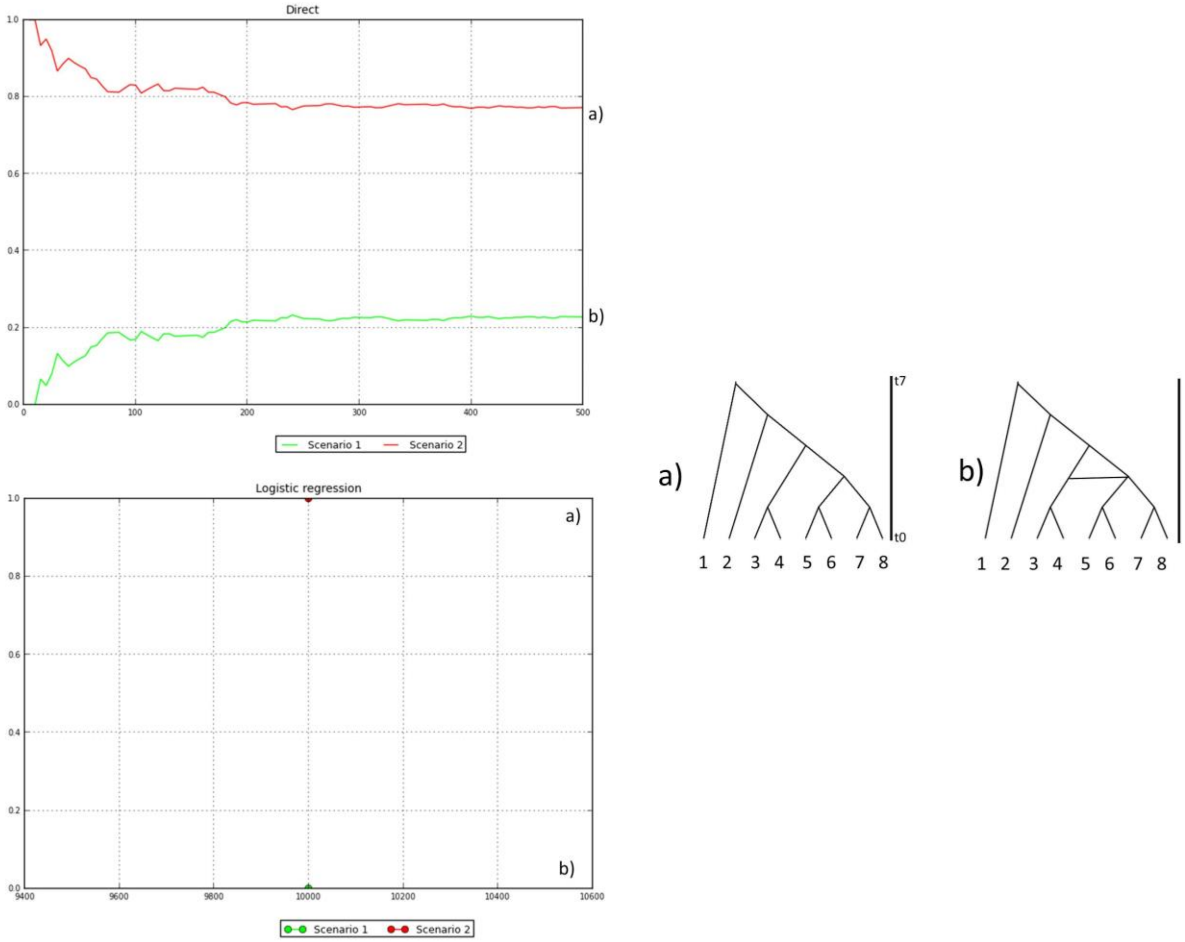

Figure S2. Posterior probabilities (Direct and Logistic) of the two previously selected domestication scenarios tested with DIYABC among eight wild and cultivated populations of *Phaseolus coccineus* in Mexico. Both scenarios reflect a paraphyletic relationship among Wild populations and a) with one contribution in time of Wild Populations into Cultivated ancestral genetic pool and b) with several contributions events in time of Wild Populations into Cultivated ancestral genetic pool

1)

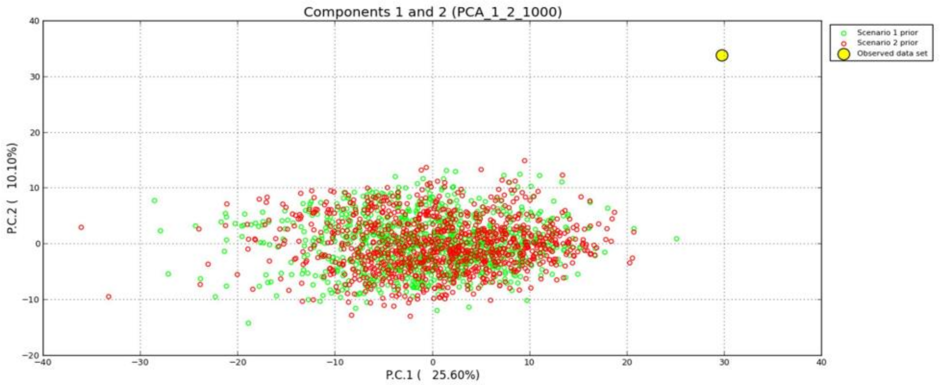

2)

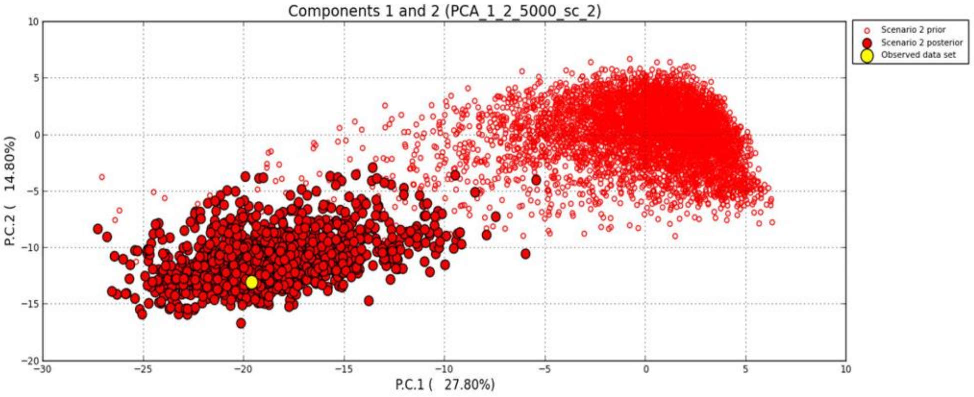

Figure S3. Principal component analyses of the a) Pre-evaluate scenario prior combinations of two selected scenarios in which paraphyletic relationship among Wild populations are represented and b) Model checking of the selected scenario indicating a single contribution in time of Wild Populations into Cultivated ancestral genetic pool

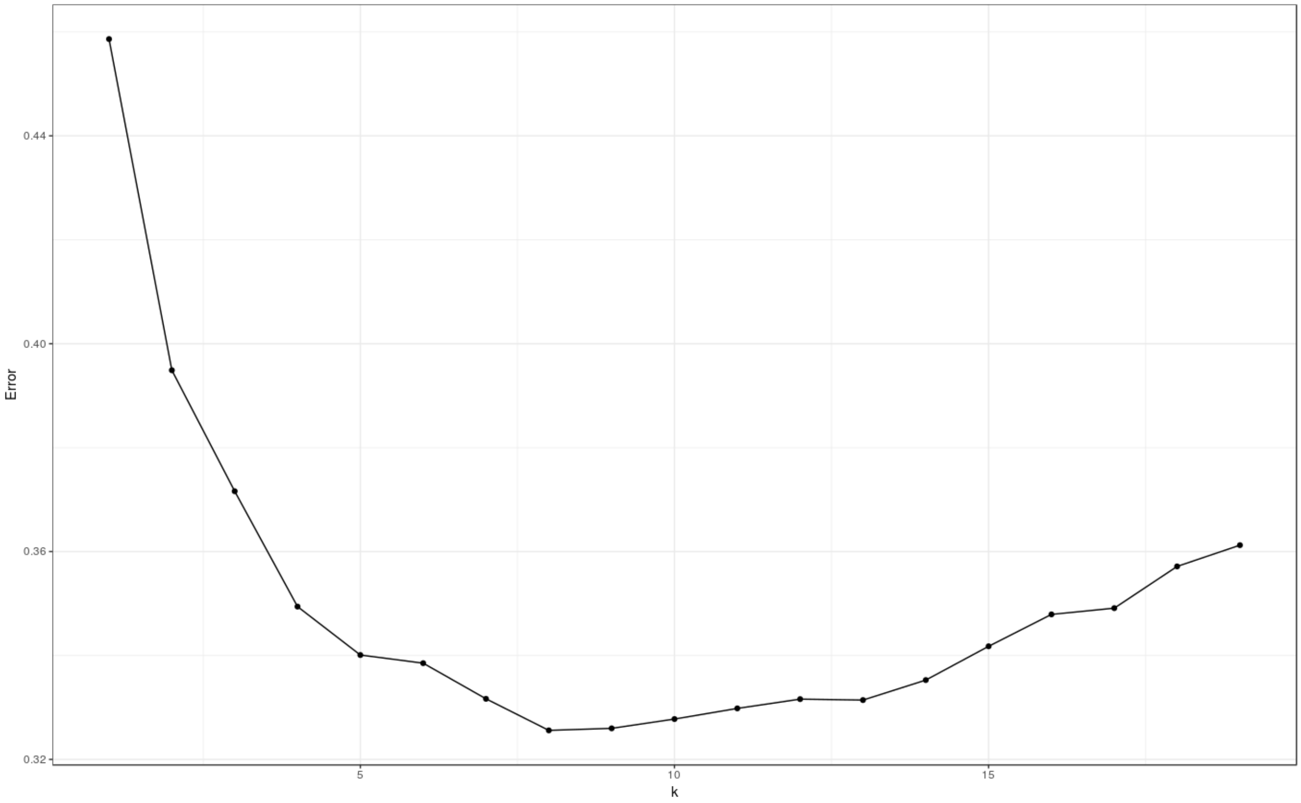

Figure S4. Cross-validation errors among *K* values using Admixture software for the *P. coccineus* data set.

Table S2. Heterozygosity and inbreeding coefficient statistics of *P. coccineus* samples grouped by population.

Genetic cluster	Population	H_O	H_E	F_{IS} (95% CI)
Cult-OV	Cuilapam	0.171	0.148	-0.152 (-0.164--0.142)
Cult-SMOCC	Blanco Tlaxcala	0.194	0.167	-0.159 (-0.168--0.150)
	Durango	0.218	0.173	-0.262 (-0.270--0.253)
	Regocijo	0.205	0.160	-0.280 (-0.293--0.273)
	Villa Unión	0.179	0.167	-0.072 (-0.083--0.063)
Cult-SUR-CH	Altotonga	0.215	0.177	-0.216 (-0.225--0.208)
	González de León	0.200	0.176	-0.135 (-0.144--0.126)
	Huautla	0.219	0.199	-0.103 (-0.112--0.095)
	Nahá	0.196	0.167	-0.171 (-0.182--0.162)
	Oxchuc	0.200	0.178	-0.124 (-0.133--0.114)
	Zicanantán	0.196	0.191	-0.029 (-0.091--0.064)
Cult-TMVB	España	0.166	0.134	-0.237 (-0.253--0.230)
	Frijol Colorado	0.193	0.191	-0.009 (-0.018-0.000)
	Orilla del Monte	0.221	0.195	-0.133 (-0.142--0.125)
	Tlalancaneca	0.199	0.190	-0.044 (-0.053--0.036)
	Puebla	0.206	0.191	-0.079 (-0.089--0.070)
Wild-SMOCC	Espinazo del Diablo	0.150	0.118	-0.274 (-0.293--0.268)
	Regocijo-S	0.165	0.141	-0.173 (-0.189--0.166)
Wild-striatus	Tres Marías	0.203	0.160	-0.267 (-0.277--0.261)
Wild-SUR-CH	Comaltepec	0.189	0.157	-0.200 (-0.231--0.207)
	San Cristobal	0.182	0.164	-0.105 (-0.125--0.102)
Wild-TMVB	Ciudad Guzman	0.169	0.176	-0.072 (-0.088--0.056)
	REPSA	0.237	0.202	-0.175 (-0.183--0.168)
	San Joaquín	0.195	0.160	-0.221 (-0.235--0.216)
	Tepoztlán	0.229	0.188	-0.217 (-0.225--0.210)
	Tlalpan	0.247	0.208	-0.184 (-0.192--0.177)

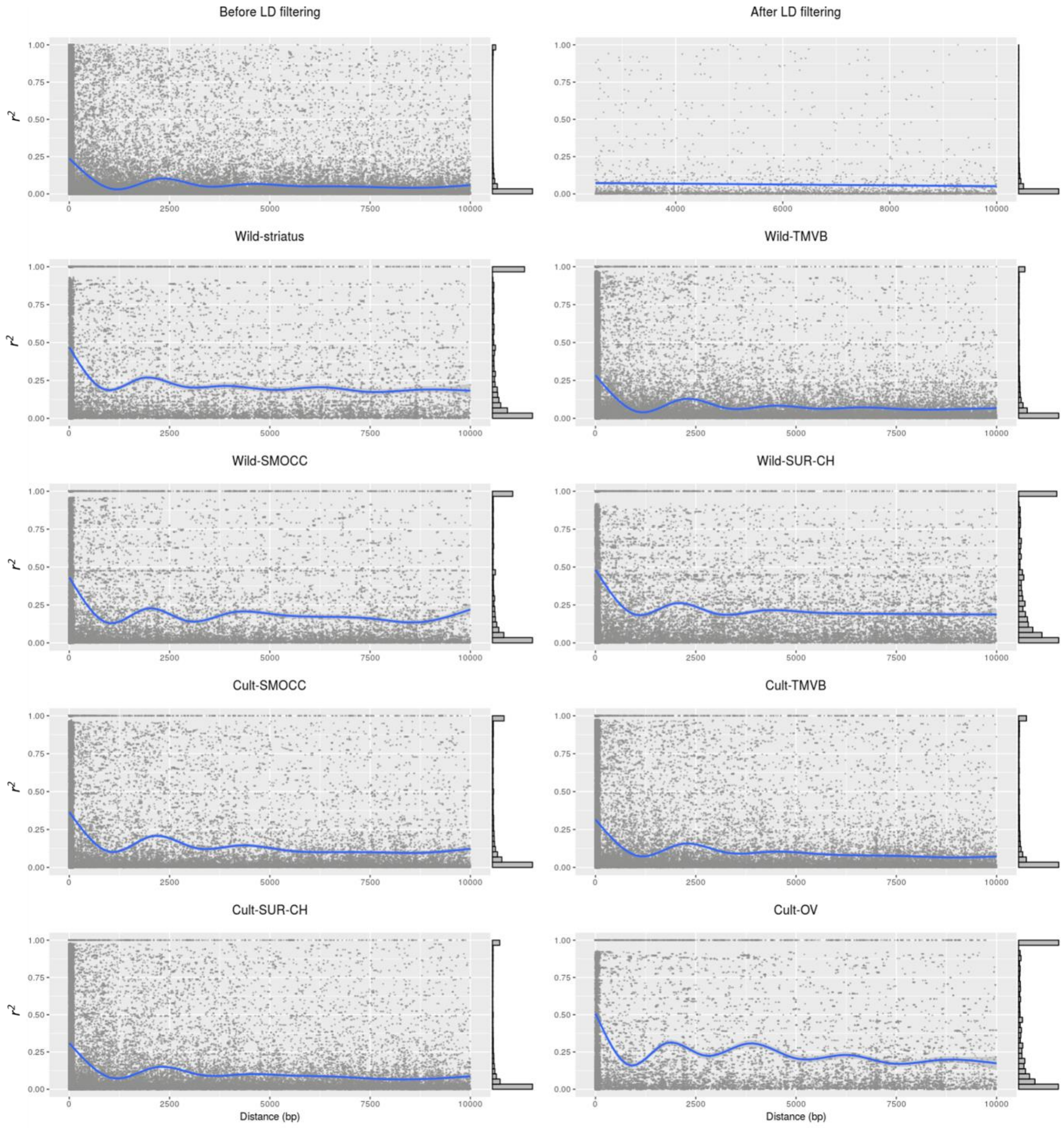

Figure S5. Pair r^2 value among SNPs located in the same chromosome separated by a maximum distance of 10 000 bp. At the right side of each plot there is a histogram showing the frequency of r^2 values.

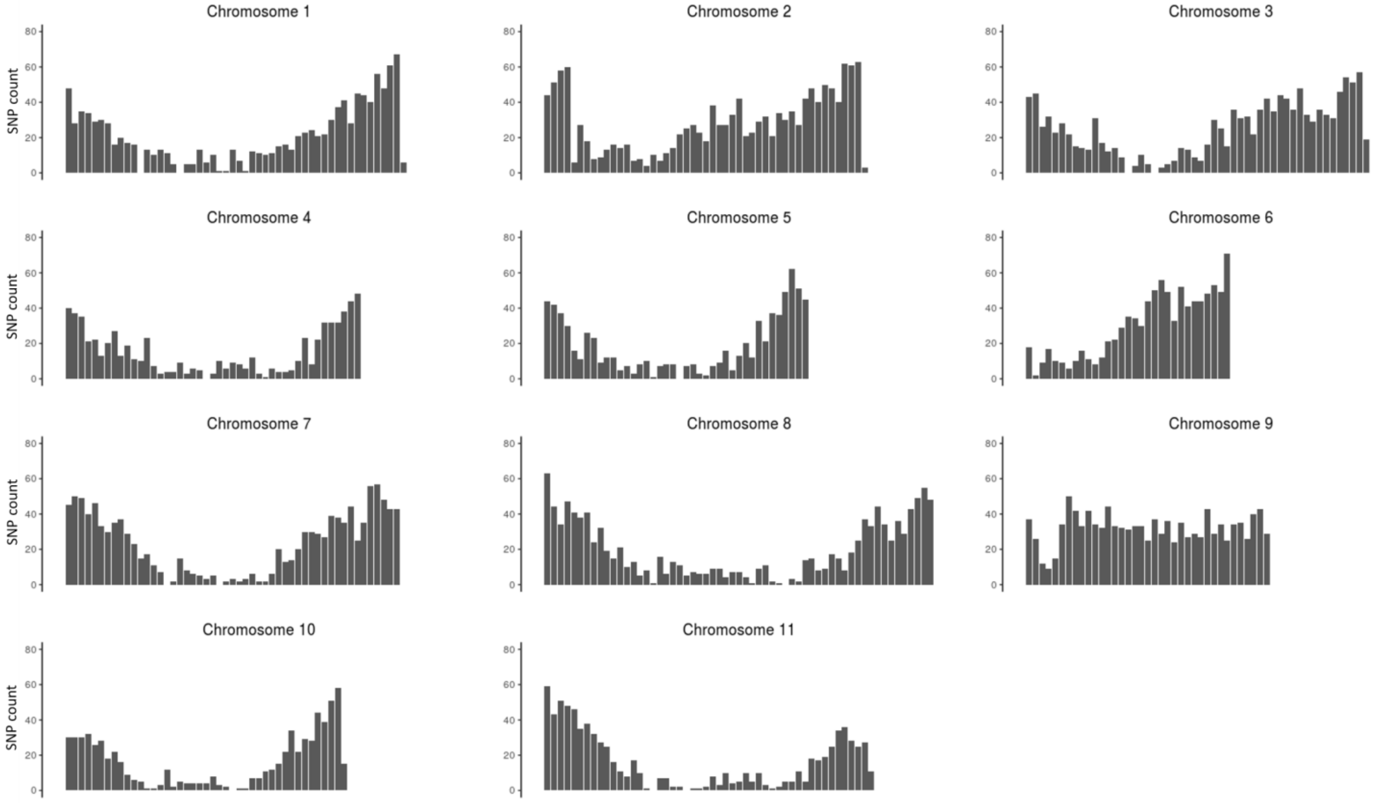

Figure S6. Distribution of 11 693 SNPs detected in *P. coccineus* (after LD filtering) present in each chromosome. Each bar represents a region of one Mb. The differences in plots sizes show the discrepancies in chromosomes lengths.

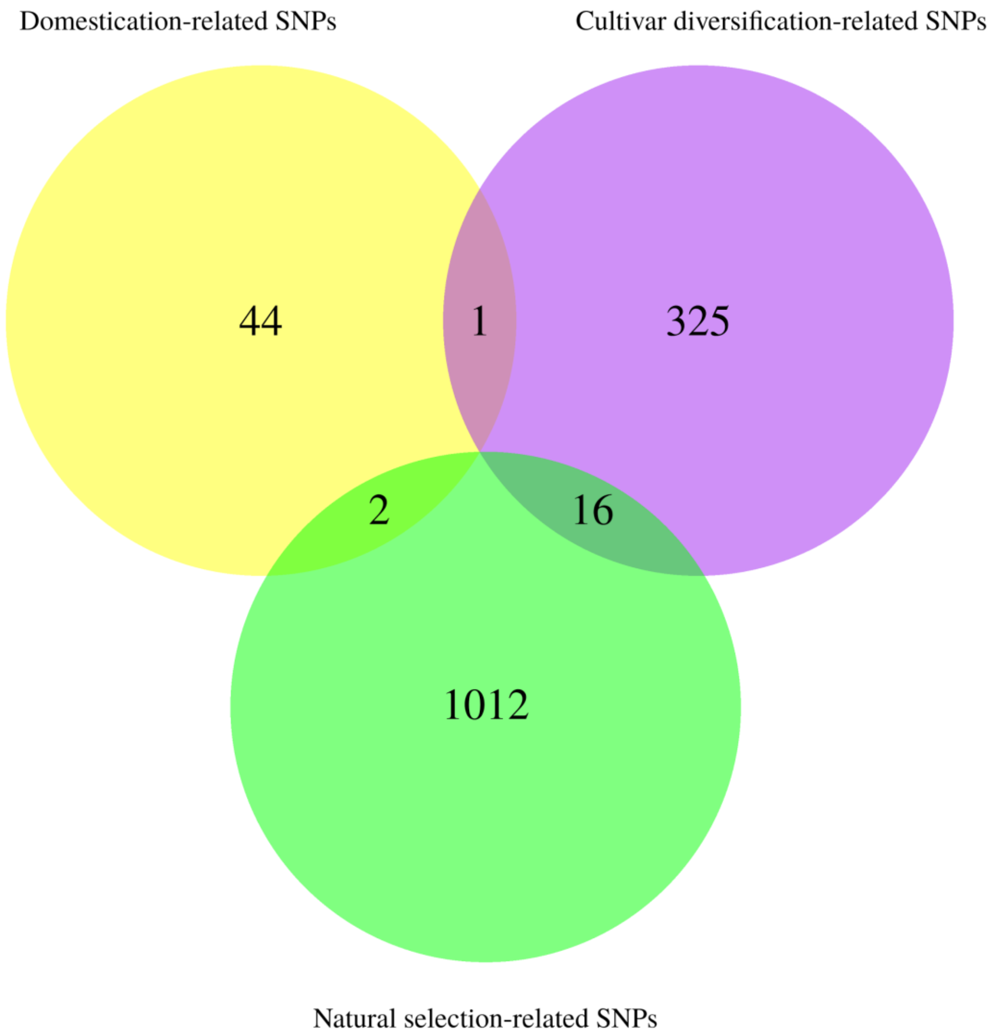

Figure S7. Venn diagram showing share candidate SNPs detected in the three PCAdapt analysis to detect natural and artificial selective pressures.

Table S3. Candidate loci identified by the pcadapt and BayeScan methods.

**Chr**	**SNP ID**	**Position**	***P. vulgaris* transcript name**	**Annotation**	**High expression tissue**	**Protein homologs *in G. max***
Domestication-related candidate						
1	S1_15176129	15176129				
1	S1_48602792	48602792	Phvul.001G232200	Phosphomethylpyrimidine kinase	Flower, buds	Glyma.11G218700
2	S1_56416681	4211050				
2	S1_79642304	27436673				
2	S1_81673246	29467615	Phvul.002G145600	TIR domain	Green and mature pods	Glyma.12G135600
2	S1_85681762	33476131				
2	S1_86298230	34092599				
3	S1_128138350	26891681				
3	S1_145377297	44130628				
6	S1_248268696	7958113				
6	S1_250412008	10101425				
6	S1_252913073	12602490				
6	S1_259648113	19337530				
6	S1_262918075	22607492				
7	S1_310062177	37774238	Phvul.007G256000	DnaJ domain	Flowers, young pods	Glyma.02G179900
7	S1_313970760	41682821				
8	S1_329571814	5525253				
8	S1_350061425	26014864				
8	S1_350157505	26110944				
8	S1_375823077	51776516				
9	S1_406871596	23162403	Phvul.009G156400	Dehydrogenase E1 component	Flower buds, flowers	Glyma.04G212100

11S1_467512979	3058827				
11S1_470314329	5860177				
11S1_472148556	7694404				
Natural selection-related candidate windows					
3S1_103319613	2072944				
3S1_108854438	7607769				
3S1_126647289	25400620				
3S1_138412433	37165764				
3S1_141480201	40233532				
3S1_143433256	42186587	Phvul.003G197500	Calmodulin binding protein-like	Root, stem	Glyma.17G092700
Cultivar diversification-related candidate loci					
1S1_45827637	45827637				
3S1_146749207	45502538				
4S1_178058430	24527352				
4S1_178200752	24669674				
4S1_178417950	24886872				
4S1_181150184	27619106				
4S1_183310744	29779666				
5S1_219843009	20351812				
5S1_220112744	20621547				
5S1_225806704	26315507				
6S1_271294756	30984173				
7S1_301822240	29534301				
9S1_388863253	5154060				
